# Cytoreductive Surgery for Extensive Intra-Abdominal and Abdominal Wall Metastases from Papillary Thyroid Carcinoma: A Case Report and Review of the Literature

**DOI:** 10.3390/jcm15135011

**Published:** 2026-06-27

**Authors:** Wadha Almohamdi, Mohsin Murshid, Saad AlHarthi, Alanoud Alghanem, Saud Almuhummadi, Abdelrazak Meliti, Hussam Bitar

**Affiliations:** 1 Department of General Surgery, King Faisal Specialist Hospital and Research Centre, Jeddah 23433, Saudi Arabia; aalghanem2@kfshrc.edu.sa (A.A.); salmuhammadi@kfshrc.edu.sa (S.A.); hbitar@kfshrc.edu.sa (H.B.); 2 Department of General Surgery, Hera General Hospital, Al Madinah Al Munawarah Rd, Makkah 24227, Saudi Arabia; mohsin.murshid90@gmail.com; 3 Department of General Surgery, King Fahad Armed Forces Hospital, Jeddah 23311, Saudi Arabia; s.a.alharthi572@kfafh.med.sa; 4 Department of Pathology, King Faisal Specialist Hospital and Research Centre, Jeddah 23433, Saudi Arabia; ameliti2014@gmail.com

**Keywords:** papillary thyroid carcinoma, intra-abdominal metastasis, distant metastasis, cytoreductive surgery, debulking surgery, case report

## Abstract

**Background:** Papillary thyroid carcinoma (PTC) is typically associated with an excellent prognosis, and distant metastases are uncommon. When present, metastases most frequently involve the lungs and bones, while intra-abdominal dissemination is exceedingly rare and poorly characterized. The optimal management of such atypical metastatic patterns, particularly the role of surgery, remains undefined. **Case Presentation:** A 69-year-old female presented eight years after total thyroidectomy, cervical lymph node dissection, and radioactive iodine therapy for PTC with progressive abdominal pain, nausea, vomiting, and painful abdominal wall and bilateral inguinal masses. Imaging demonstrated extensive metastatic disease involving the anterior abdominal wall, gastrohepatic ligament, pelvis, retroperitoneum, diaphragm, urinary bladder, uterus, and inguinal lymph nodes, which was confirmed on biopsy as metastatic PTC. Serum thyroglobulin was markedly elevated at 473 µg/L. Given the bulky, symptomatic, and progressive disease refractory to prior radioactive iodine therapy, a multidisciplinary tumor board recommended cytoreductive surgery with palliative intent. The patient underwent resection of eleven major metastatic deposits over five hours without intraoperative complications, achieving complete macroscopic clearance of intra-abdominal disease. Histopathology confirmed metastatic PTC in all specimens. Postoperative serum thyroglobulin declined to 107 µg/L following surgery and adjuvant radioactive iodine therapy, and the patient reported complete resolution of abdominal symptoms at follow-up. **Conclusions:** This case highlights the potential role of cytoreductive surgery as a palliative strategy in carefully selected patients with advanced intra-abdominal metastatic PTC. Such rare presentations underscore the importance of long-term surveillance and multidisciplinary decision-making. Further case accumulation is needed to better define optimal management strategies and patient selection criteria in this setting.

## 1. Introduction

Papillary thyroid carcinoma (PTC) is the most common subtype of differentiated thyroid cancer and is generally associated with an excellent prognosis following appropriate surgical and adjuvant management [1]. Most patients present with localized disease, often as an asymptomatic thyroid nodule or cervical lymphadenopathy, and long-term survival exceeds 90% in the absence of distant metastases [2]. While regional lymph node involvement is common, distant metastases are relatively uncommon and typically indicate advanced disease with a less favorable prognosis [3].

The lungs and bones represent the most frequent sites of distant metastases in PTC [3,4]. In contrast, involvement of intra-abdominal organs—including the liver, pancreas, gastrointestinal tract, abdominal wall, and genitourinary structures—is exceedingly rare, with reported incidences of less than 1% in large series [5,6,7,8]. In a large multicenter study of 38,772 differentiated thyroid cancer patients, unusual metastases to sites beyond the lung, bone, and brain were identified in only 0.06% of cases, with the kidney, liver, and pancreas representing the most frequently affected unusual sites [8]. The majority of such metastases follow a metachronous pattern, often presenting more than nine years after the primary diagnosis [8]. The available literature on these atypical metastatic patterns consequently remains largely limited to isolated case reports and small case series, leaving significant gaps in the understanding of their clinical behavior, optimal management strategies, and outcomes [5,6,7,8].

The role of surgical intervention in patients with extensive intra-abdominal metastatic disease remains particularly unclear. While surgery is well established in the management of locoregional PTC, its application in disseminated metastatic settings—especially for symptom control or cytoreduction—has not been well defined, and current guidelines provide limited direction for these rare clinical scenarios [9,10].

We report a rare case of extensively disseminated intra-abdominal and abdominal wall metastases from PTC presenting eight years after primary treatment, in which the patient underwent extensive cytoreductive surgery for symptomatic, progressive disease. This report aims to highlight the clinical presentation, surgical decision-making, and potential role of debulking surgery in selected patients with advanced metastatic PTC, accompanied by a narrative review of the relevant literature.

## 2. Case Presentation

A 69-year-old female with a history of papillary thyroid carcinoma (PTC)—notable for its unusually delayed and anatomically atypical pattern of distant metastasis—presented eight years after initial treatment with a four-month history of progressive abdominal pain, nausea, vomiting, and painful paraumbilical swelling, accompanied by gradually enlarging bilateral groin masses. Initial treatment consisted of total thyroidectomy, left cervical lymph node dissection, and two courses of radioactive iodine (RAI) therapy. There were no symptoms suggestive of locoregional thyroid recurrence, and neck examination was unremarkable.

Abdominal examination revealed a firm, tender paraumbilical mass measuring approximately 5 × 4 cm and bilateral solid inguinal masses. Laboratory evaluation demonstrated a markedly elevated serum thyroglobulin level of 473 µg/L (anti-thyroglobulin antibody status was negative, confirming the reliability of this measurement) and an elevated CA-125 of 65.7 U/mL. Neck ultrasound showed no evidence of locoregional recurrence. Contrast-enhanced CT of the abdomen and pelvis revealed multiple metastatic deposits, including superficial solid lesions in the anterior abdominal wall at the umbilical region, a right pelvic lesion, bilateral enlarged inguinal lymph nodes, and additional lesions involving the gastrohepatic ligament and retroperitoneum (Figure 1 and Figure 2). Ultrasound-guided biopsy of the paraumbilical mass confirmed metastatic PTC.

Whole-body radioiodine (I-123) scintigraphy demonstrated abnormal uptake within bilateral inguinal lymph nodes, paraumbilical subcutaneous masses, a right pelvic cystic lesion, subdiaphragmatic metastases, and multiple pleural-based and pulmonary lesions (Figure 3), confirming heterogeneous but widespread radioiodine avidity. Despite prior thyroidectomy, two courses of RAI therapy, and TSH suppression, the patient experienced progressive disease with severe, refractory abdominal pain requiring multiple hospital admissions, establishing a clinical picture of bulky, symptomatic, and partially radioiodine-refractory metastatic PTC.

The case was reviewed by a multidisciplinary tumor board comprising endocrine surgeons, surgical oncologists, endocrinologists, nuclear medicine physicians, radiologists, and pathologists. Given the severity of the patient’s symptoms and the risk of local complications from enlarging abdominal and pelvic masses, cytoreductive surgery was recommended with palliative intent to reduce tumor burden, alleviate symptoms, and improve quality of life. Systemic therapy was considered but deferred given the partial radioiodine avidity of the disease, the dominance of locoregional symptom burden over systemic disease progression, and the absence of targetable mutations on available molecular profiling. The patient was counseled regarding the extent of systemic disease, the presence of non-resectable pulmonary metastases, and the non-curative nature of the procedure.

Exploratory laparotomy revealed a large umbilical mass with bluish discoloration consistent with cutaneous vascular infiltration, areas of impending ulceration, and involvement of the umbilical stalk, anterior rectus sheath, and part of the right rectus muscle. The abdominal wall mass was excised en bloc together with the umbilical stalk, involved fascia, and the affected portion of the right rectus muscle. The falciform and round ligaments, both containing multiple metastatic nodules, were also excised.

Further exploration confirmed extensive metastatic involvement of the gastrohepatic ligament, caudate lobe region, retroperitoneum, right adnexa, bilateral inferior mesenteric lymph nodes, bladder wall, uterine serosa, right hemidiaphragm, bilateral inguinal lymph nodes, and multiple small bowel mesenteric nodules. Particular attention was directed toward a large gastrohepatic ligament mass extending superiorly toward the gastroesophageal junction and involving the lesser curvature and posterior gastric wall. The lesion was carefully dissected from the liver, pancreas, splenic artery, and celiac axis. Although it partially involved the caudate lobe and encased the left gastric artery, complete mobilization was achieved while preserving the splenic artery. The left gastric artery was divided at its origin, and en bloc partial gastrectomy with limited lymphadenectomy was performed. The gastric staple line was reinforced with oversewn sutures, and intraoperative esophagogastroduodenoscopy confirmed an intact anastomosis without evidence of leakage.

Within the pelvis, a large partially cystic right adnexal mass and a retroperitoneal lesion overlying the gonadal vessels were excised. Two bladder wall nodules and a lesion located between the bladder and uterus were resected, along with metastatic deposits adjacent to the inferior mesenteric artery and additional contralateral lesions. Bilateral inguinal lymphadenectomy was subsequently performed for the matted, multinodal metastatic disease identified preoperatively—an anatomically unusual site of PTC metastasis that warranted formal nodal clearance given the burden of disease.

In total, eleven major metastatic deposits or nodal groups were resected: the abdominal wall mass, gastrohepatic ligament mass, gastric lesion, right adnexal mass, right retroperitoneal mass, bilateral inferior mesenteric lymph node metastases, bladder lesion, uterine lesion, right diaphragmatic lesion, and bilateral inguinal lymph node metastases. The procedure lasted approximately five hours with an estimated blood loss of 300 mL; no transfusion was required, and no intraoperative complications occurred. At completion, no gross residual intra-abdominal or pelvic disease was identified, corresponding to complete macroscopic cytoreduction. Known thoracic metastatic disease was deferred from surgical management, as these lesions were not amenable to resection and were planned for subsequent RAI therapy and TSH suppression.

Two closed-suction drains were placed—one adjacent to the gastric resection bed within the gastrohepatic region and one within the pelvis. The postoperative course was uneventful, and the patient was discharged on postoperative day seven with marked symptomatic improvement, reporting complete resolution of abdominal pain, nausea, and vomiting and tolerating oral intake without difficulty.

Histopathological examination confirmed metastatic PTC in all resected specimens. Positivity of both the right adnexal mass and uterine lesion for metastatic PTC excluded a primary gynecologic malignancy, and the elevated CA-125 was attributed to extensive peritoneal and pelvic tumor involvement. Morphologically, both the primary tumor and metastatic deposits shared similar cytological features: papillary architecture with well-formed fibrovascular cores, nuclear crowding, overlapping, elongation, prominent nuclear grooves, and conspicuous nucleoli. Focal oncocytic cytoplasmic change and rare psammoma bodies were present, without increased mitotic activity, a solid growth pattern, or tumor necrosis (Figure 4). Immunohistochemistry demonstrated positivity for thyroglobulin, TTF-1, and PAX-8, confirming a thyroid follicular epithelial origin; WT-1 and p53 were negative (Figure 5). BRAF/RAS mutation status was not available at the time of reporting; this information, if obtained, would further inform the understanding of radioiodine refractoriness in this case. Ten of ten retrieved lymph nodes were positive for metastatic disease. The gastrohepatic ligament specimen demonstrated tumor within less than 1 mm of the inked surgical margin, though no gross residual intra-abdominal disease was evident at operative completion.

Following recovery, the patient underwent adjuvant I-131 therapy targeting residual thoracic disease. Post-treatment scintigraphy demonstrated interval resolution of previously iodine-avid abdominal and inguinal lesions. Persistent low-grade uptake remained within right-sided pleural and pulmonary metastases, while additional contralateral pulmonary nodules and multiple intrathoracic lymph nodes were iodine-negative, confirming a pattern of heterogeneous radioiodine sensitivity with partially refractory disease. A newly detected iodine-avid right breast lesion was investigated with dedicated breast imaging and biopsy, which confirmed a benign etiology.

Serum thyroglobulin declined substantially from 473 µg/L preoperatively to 107 µg/L following cytoreductive surgery and adjuvant RAI therapy, representing a meaningful biochemical response. Follow-up CT demonstrated no recurrent bulky disease within previously resected abdominal regions and stable right-sided pulmonary metastases.

At 12-month follow-up, the patient remained clinically well with sustained symptomatic benefit. From an oncologic standpoint, however, surveillance imaging demonstrated progression of residual abdominal deposits, including perihepatic peritoneal lesions measuring up to 4.4 × 3.6 cm, retroperitoneal lymphadenopathy, and a multiloculated left adnexal cystic lesion measuring 4.5 × 3.1 cm, while pulmonary disease remained radiologically stable. An anterior abdominal wall hernia containing a portion of the transverse colon was identified on follow-up imaging and is being managed accordingly.

### Review of Literature

A narrative review of the published literature was performed to contextualize the present case. PubMed was searched on 11 June 2026 using combinations of the following terms: “papillary thyroid carcinoma,” “differentiated thyroid carcinoma,” “abdominal metastasis,” “intra-abdominal metastasis,” “liver metastasis,” “hepatic metastasis,” “liver neoplasms,” “pancreatic metastasis,” “pancreatic neoplasms,” “abdominal wall metastasis,” “distant metastasis,” “unusual metastasis,” “rare metastasis,” “inguinal lymphadenopathy,” “inguinal lymph node,” “hepatic hemorrhage,” “uterine metastasis,” “bladder metastasis,” and “diaphragm metastasis.” Only English-language reports describing intra-abdominal or atypical extra-cervical metastases from papillary thyroid carcinoma were included, with no date restrictions applied. The full search and screening process is detailed in the PRISMA flow diagram (Figure 6). Two additional cases were identified through reference list screening. A total of 138 records were screened, of which 15 were included in the final review. The available evidence was largely confined to individual case reports and small case series. The present case is not included in this review.

The available literature highlights the remarkable heterogeneity of intra-abdominal metastatic PTC with respect to metastatic sites, timing of presentation, treatment strategies, and clinical outcomes. The pancreas and liver appear to be the most frequently reported intra-abdominal sites, although both remain exceedingly uncommon compared with pulmonary and osseous metastases [2,11,12,13,14,15,16,17,18,19]. Unusual pelvic and genitourinary involvement has also been described, including metastasis to the uterine corpus [20] and bilateral inguinal lymph nodes [21], as well as urinary bladder involvement [22]—sites that are particularly relevant to the pattern of dissemination observed in the present case. Several reports describe metastases presenting many years after initial thyroid cancer treatment, with documented intervals ranging from three to eleven years, emphasizing the prolonged natural history of differentiated thyroid carcinoma and its capacity for late recurrence [12,14,18,19,23]. Synchronous intra-abdominal metastases detected at or prior to the primary thyroid cancer diagnosis have also been described, often mimicking primary pancreatic or other abdominal neoplasms and presenting significant diagnostic challenges [2,11,13,20,21,22,24].

Management strategies reported in the literature vary considerably and are influenced by disease extent, symptom burden, and iodine avidity. Radioactive iodine therapy remains an important treatment modality in patients with iodine-avid disease, with documented radiologic and biochemical responses in selected cases [15,16,22]. Surgical resection has been employed in selected patients with isolated or symptomatic lesions, with durable disease control reported particularly when complete resection was achieved in single-organ disease [13,14,18,23]. However, pancreatic resection was deferred in favor of systemic therapy in patients with multi-organ involvement [12,19]. Tyrosine kinase inhibitor therapy has been employed in patients with radioiodine-refractory disease—lenvatinib achieved a significant biochemical response in Yang et al. [2], achieving a serum thyroglobulin decline from 313.60 to 15.84 µg/L over eight months, while sorafenib was associated with stable disease in Tramontin et al. [14]. However, lenvatinib failed to prevent disease progression in Thomasson et al. [24], highlighting the variable response to systemic therapy in advanced disease. Outcomes appear considerably less favorable in patients with disseminated or radioiodine-refractory multi-organ disease, with disease-specific mortality documented in reported cases [19,21,24], underscoring the poor prognosis associated with extensively disseminated or treatment-refractory presentations.

Despite the increasing number of reported cases, the literature remains insufficient to establish evidence-based recommendations regarding the role of surgery in extensive intra-abdominal metastatic PTC. Most published reports describe solitary or oligometastatic disease involving a single organ, whereas cases of multi-organ abdominal and pelvic involvement comparable to the present case are exceptionally rare. The most comparable report is that of Yang et al. [2], in which synchronous metastases to the pancreas, liver, diaphragm, parotid glands, salivary glands, gluteus maximus, and multiple other sites were managed with an aggressive multimodal approach including surgery, RAI, and lenvatinib—achieving serum thyroglobulin decline from 313.60 to 15.84 µg/L and clinical stability at the time of reporting, though with radioiodine-refractory disease confirmed on post-ablation scintigraphy. Management decisions in such rare presentations therefore continue to rely on multidisciplinary assessment and individualized patient-centered decision-making. A summary of published cases is presented in Table 1.

## 3. Discussion

Papillary thyroid carcinoma is the most prevalent endocrine malignancy worldwide, accounting for approximately 80% of all thyroid cancers, and its incidence has risen markedly over recent decades, largely attributable to increased detection of subclinical disease through advanced imaging [1]. Despite this rising incidence, disease-specific mortality has remained relatively stable, reflecting the generally indolent behavior and excellent long-term prognosis associated with localized disease [1]. However, the development of distant metastases represents a critical inflection point in the clinical course of PTC. Recent data from a large national cohort demonstrate that radioiodine refractoriness and involvement of multiple metastatic sites are the strongest independent predictors of disease-specific mortality in patients with metastatic differentiated thyroid cancer, with multi-organ involvement associated with substantially worse survival outcomes [25]. These findings underscore the fundamental shift in prognosis that accompanies distant dissemination and provide important context for the clinical scenario described in this report.

This case represents one of the most extensively disseminated intra-abdominal presentations of metastatic PTC reported in the literature. The simultaneous involvement of the abdominal wall, gastrohepatic ligament, stomach, retroperitoneum, diaphragm, bladder, uterus, adnexa, bilateral inferior mesenteric lymph nodes, and bilateral inguinal lymph nodes represents a degree of disease burden that substantially exceeds the oligometastatic patterns described in most previously reported cases [11,12,13,14,15,16,17,18,19,23,24]. The most comparable published case is that of Yang et al. [2], in which synchronous metastases to the pancreas, liver, diaphragm, parotid glands, salivary glands, gluteus maximus, and multiple other sites were documented at initial presentation—yet even this case did not involve the degree of pelvic, genitourinary, and abdominal wall dissemination observed in the present patient. Disease recurrence occurring eight years after initial thyroidectomy and RAI therapy further highlights the capacity of differentiated thyroid carcinoma for very late metastatic presentation and reinforces the importance of long-term surveillance in all patients with a prior history of PTC [8].

The diagnostic workup in this case deserves particular attention. A markedly elevated serum thyroglobulin level of 473 µg/L with negative anti-thyroglobulin antibodies, widespread radioiodine-avid lesions on I-123 scintigraphy, and biopsy-confirmed metastatic PTC at the paraumbilical mass together established the diagnosis with confidence. The elevated CA-125 level initially raised concern for a primary gynecologic malignancy. However, histopathological positivity for thyroglobulin, TTF-1, and PAX-8 in both the adnexal and uterine specimens confirmed that these lesions represented metastatic PTC rather than a gynecologic primary. This is an important diagnostic pitfall: in patients with known PTC and an elevated CA-125 level, pelvic metastatic disease should be considered before attributing the finding to a gynecologic malignancy [12,24].

The decision to proceed with surgery was informed by several factors. The patient had severe, refractory abdominal pain requiring multiple hospital admissions, alongside progressively enlarging abdominal wall and inguinal masses that were significantly affecting her quality of life. Prior RAI therapy and TSH suppression had failed to control the intra-abdominal disease burden despite partial iodine avidity. Systemic kinase inhibitor therapy was considered but deferred, given the partial radioiodine avidity of the disease, the predominance of locoregional symptoms over systemic progression, and the absence of targetable mutations on available molecular profiling. In this setting, cytoreductive surgery was considered the most direct and immediate option for symptom relief and local disease control [10,13,14,18,23].

The decision to defer systemic therapy in the present case warrants further discussion in the context of current management guidelines and evolving treatment strategies for advanced differentiated thyroid carcinoma. The 2025 American Thyroid Association guidelines recommend molecular biomarker testing as a prerequisite step before initiating systemic therapy in patients with radioiodine-refractory progressive DTC, to identify actionable oncogenic driver alterations that may guide treatment selection [10]. In the absence of targetable mutations, lenvatinib and sorafenib remain the recommended first-line multitargeted tyrosine kinase inhibitors for progressive, symptomatic radioiodine-refractory DTC, with lenvatinib generally preferred based on the superior progression-free survival demonstrated in the phase III SELECT trial [10,26]. Cabozantinib is recommended as a second-line option following failure of or intolerance to initial TKI therapy [10]. For patients harboring specific actionable alterations, selective targeted agents are now preferred, including selpercatinib or pralsetinib for RET fusion-positive disease, and larotrectinib or entrectinib for NTRK fusion-positive disease [10,27]. In the present case, systemic therapy was deferred for several reasons consistent with current guideline principles. The disease demonstrated partial radioiodine avidity at the time of surgical decision-making, and molecular profiling did not identify a targetable oncogenic driver alteration. The dominant clinical problem was locoregional symptom burden—severe, refractory abdominal pain, progressive mass effect, and risk of local complications—rather than systemic disease progression, for which cytoreductive surgery offered more immediate and direct benefit. The ATA 2025 guidelines acknowledge that not all patients with advanced thyroid cancer require immediate initiation of systemic therapy, particularly when locoregional interventions can address the dominant source of morbidity [10]. Nevertheless, systemic therapy remains an important consideration in the ongoing management of this patient, particularly given the heterogeneous radioiodine sensitivity and documented progression of residual peritoneal and pelvic deposits at 12-month follow-up.

Current guidelines offer limited direction for surgery in disseminated intra-abdominal metastatic PTC, given the rarity of such presentations and the lack of prospective data [9,10]. Most published experience is limited to isolated metastasectomy for single-organ disease and does not directly apply to patients with the extent of multi-organ involvement seen in this case. Nevertheless, the broader rationale for cytoreductive surgery—achieving meaningful palliation through tumor burden reduction in patients with symptomatic, progressive disease—provided a reasonable basis for the operative decision [13,14,18,23].

Given the absence of formal selection criteria for this rare clinical scenario, the following framework is proposed based on available published experience and the authors’ observations from the present case. Patient selection should be individualized and guided by multidisciplinary assessment [10]. Symptom burden is perhaps the most important consideration—surgery is most justifiable when bulky, progressive disease is causing significant, refractory morbidity that cannot be adequately controlled by non-surgical means, as demonstrated in several published metastasectomy reports [13,18,23]. Performance status and expected survival must be carefully evaluated, as the physiological demands of extensive cytoreduction are only appropriate in patients with adequate functional reserve [10]. The extent of extra-abdominal disease is a critical determinant—the presence of unresectable systemic metastases shifts operative intent firmly toward palliation rather than oncological control, and this must be clearly communicated to the patient before surgery [10]. Radioiodine sensitivity should be assessed prior to any surgical decision, as patients with predominantly iodine-avid disease may achieve adequate disease control through RAI therapy alone, making surgery unnecessary [8,10]. Conversely, in patients with partially or fully radioiodine-refractory disease and dominant locoregional symptoms, surgery may offer the most direct and immediate prospect of symptomatic relief [13,17]. The availability and suitability of systemic therapies—including TKI agents and, where applicable, selective targeted therapies—should be evaluated both as alternatives and as adjuncts to surgery, with sequencing discussed within the multidisciplinary team before any operative decision is made [10,26]. Finally, patient counseling should clearly address the palliative rather than curative nature of the procedure, with realistic expectations regarding oncological outcomes established before surgery.

From a technical perspective, this case shows that extensive multi-organ cytoreduction is feasible in selected patients with metastatic PTC, even when disease involves the gastrohepatic ligament, stomach, pelvic viscera, and bilateral inguinal nodal basins simultaneously. Complete macroscopic cytoreduction was achieved across all intra-abdominal and pelvic sites. The only area of close margin was the gastrohepatic ligament specimen, where tumor came within less than 1 mm of the resection margin. Performing en bloc partial gastrectomy while preserving the splenic artery and celiac axis illustrates the surgical complexity that may be required to achieve adequate cytoreduction in this setting. Bilateral inguinal lymphadenectomy, which is not a typical component of thyroid cancer surgery, was included because of the burden of matted multinodal disease and its direct contribution to the patient’s groin symptoms.

The postoperative course was uncomplicated, and early outcomes were favorable. The patient was discharged on postoperative day seven, reported complete resolution of abdominal pain, nausea, and vomiting, and serum thyroglobulin declined from 473 µg/L preoperatively to 107 µg/L following surgery and adjuvant I-131 therapy, indicating a meaningful biochemical response. Post-treatment scintigraphy confirmed resolution of previously iodine-avid abdominal and inguinal lesions. These findings confirm that cytoreductive surgery achieved its primary palliative goal of symptom relief and local disease control. However, at 12-month follow-up, surveillance imaging demonstrated progression of residual peritoneal and pelvic deposits, with new perihepatic lesions and a multiloculated left adnexal cystic lesion, while pulmonary disease remained stable. This oncologic trajectory—meaningful symptomatic and biochemical response followed by documented disease progression within one year—illustrates the fundamental limitation of surgery as a standalone strategy in extensively disseminated, partially radioiodine-refractory PTC. Surgery in this context provided palliation, not disease control, and the subsequent progression underscores the ongoing need for systemic therapy in the long-term management of such patients.

It is important to note that this report documents feasibility and symptomatic benefit in a single patient and does not establish a survival advantage, superiority over systemic therapy, or a general indication for cytoreductive surgery in metastatic PTC. When operative intervention is considered, it should be undertaken with clearly defined palliative goals, within a multidisciplinary framework, and with honest patient counseling about the non-curative nature of the procedure.

Continued reporting of similar cases remains important. Building on clinical experience will help define better patient selection criteria, clarify how surgery should be sequenced alongside RAI and systemic therapies, and improve our understanding of the natural history of extensive intra-abdominal metastatic PTC.

### Future Perspectives: Artificial Intelligence in Thyroid Cancer Diagnosis

The growing role of artificial intelligence in thyroid oncology represents an important future perspective for the diagnosis and management of thyroid malignancies, including rare and atypical presentations such as the case described here. Current AI-driven computer-aided diagnosis systems applied to thyroid ultrasound have demonstrated overall sensitivity and specificity of approximately 88% and 81%, respectively, for distinguishing benign from malignant nodules, with more advanced multimodal platforms achieving diagnostic accuracy exceeding 90% [28]. A key challenge in thyroid nodule evaluation is inter-operator variability in ultrasound interpretation—a limitation that AI-assisted standardization directly addresses by providing objective, reproducible lesion characterization [28]. Emerging platforms such as the ANTHEM framework integrate ultrasound imaging with genomic profiles and digital pathology in a multimodal diagnostic pipeline, moving beyond conventional two-dimensional image interpretation toward three-dimensional volumetric analysis and personalized risk stratification [28]. In the context of advanced and metastatic PTC, where delayed and atypical presentations pose significant diagnostic challenges, AI tools capable of integrating multimodal clinical, imaging, and molecular data hold particular promise for earlier detection, improved risk stratification, and more informed treatment decision-making.

## 4. Conclusions

Extensive intra-abdominal dissemination of papillary thyroid carcinoma is exceedingly rare and presents significant management challenges in the absence of established guidelines. This case demonstrates that cytoreductive surgery may represent a feasible and symptomatically beneficial palliative option in carefully selected patients when disease burden is causing significant morbidity and treatment goals are clearly defined. Treatment decisions should be individualized within a multidisciplinary framework, and further case accumulation is needed to better define patient selection criteria and optimal management strategies.

## Figures and Tables

**Figure 1:**
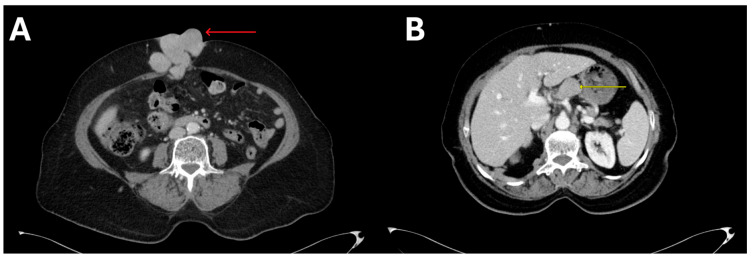
Contrast-enhanced CT demonstrating metastatic deposits: (**A**) anterior abdominal wall soft tissue lesion measuring 5.2 × 3.9 cm (red arrow), and (**B**) exophytic lesion adherent to the pancreatic body measuring 2.4 × 3.5 cm (yellow arrow).

**Figure 2:**
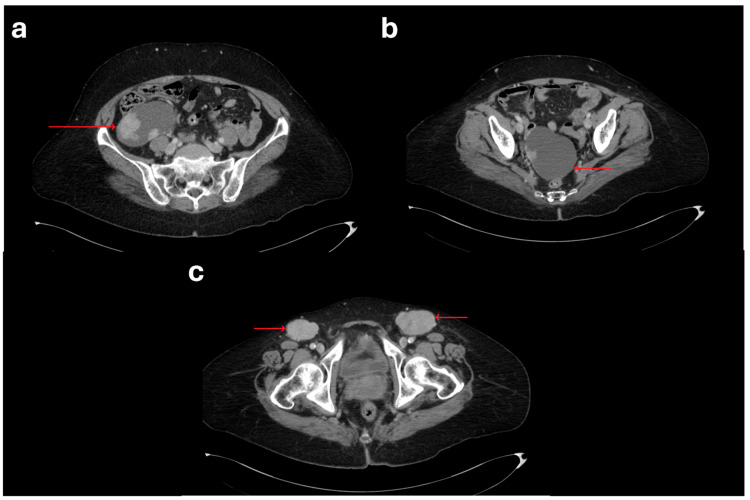
Contrast-enhanced CT demonstrating pelvic and inguinal metastatic lesions: (**a**) right pelvic cystic lesion with an intramural nodule measuring 8.1 × 6.4 cm; (**b**) second right pelvic cystic lesion with an intramural nodule measuring 8.4 × 4.7 cm; and (**c**) bilateral inguinal solid lesions measuring 4.2 × 2.9 cm on the right and 5.3 × 3.1 cm on the left (red arrows).

**Figure 3:**
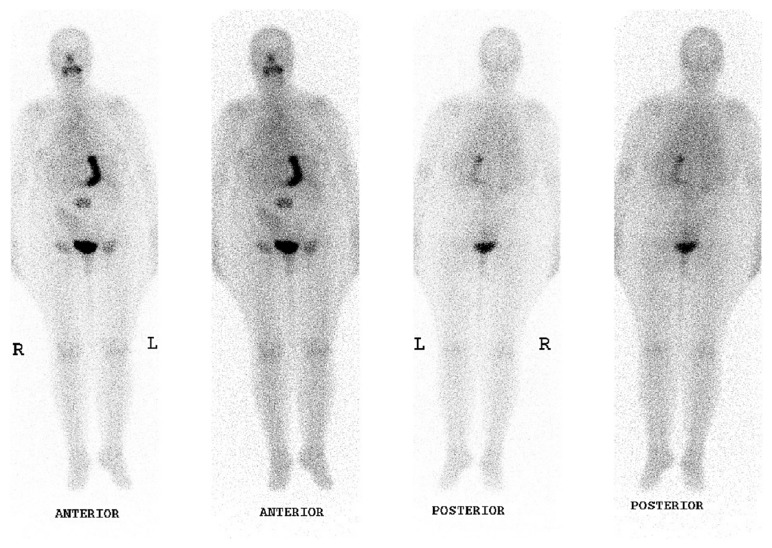
I-123 whole-body scintigraphy demonstrating multiple foci of abnormal tracer uptake, including bilateral pleural and pulmonary metastases, bilateral inguinal lymph nodes, paraumbilical subcutaneous deposits, and a right pelvic cystic lesion with an avid solid component, consistent with widespread subdiaphragmatic metastatic disease.

**Figure 4:**
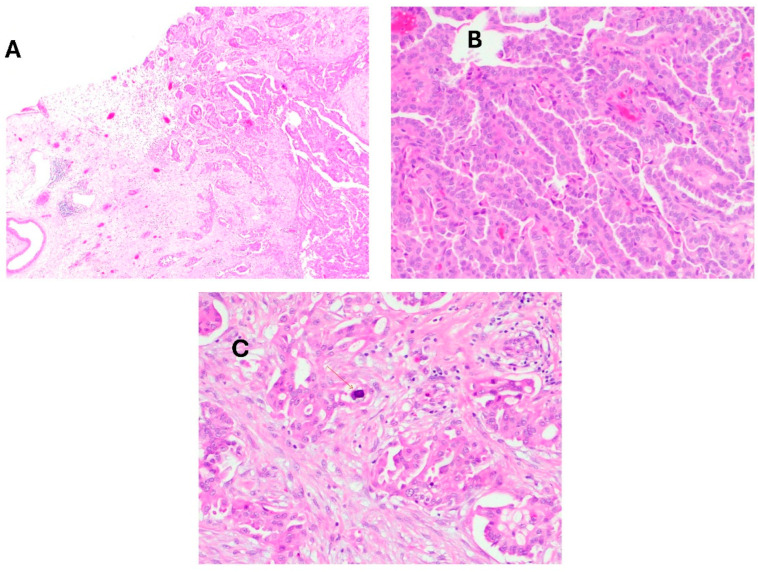
Histopathological features on H&E staining: (**A**) low-power view (50×) demonstrating predominantly papillary architecture with well-formed fibrovascular cores lined by cuboidal to low-columnar epithelial cells; (**B**) medium-power view (100×) showing nuclear overlapping, crowding, nuclear grooves, and inconspicuous nucleoli; and (**C**) medium-power view (100×) highlighting rare psammoma bodies associated with the epithelial neoplasm.

**Figure 5:**
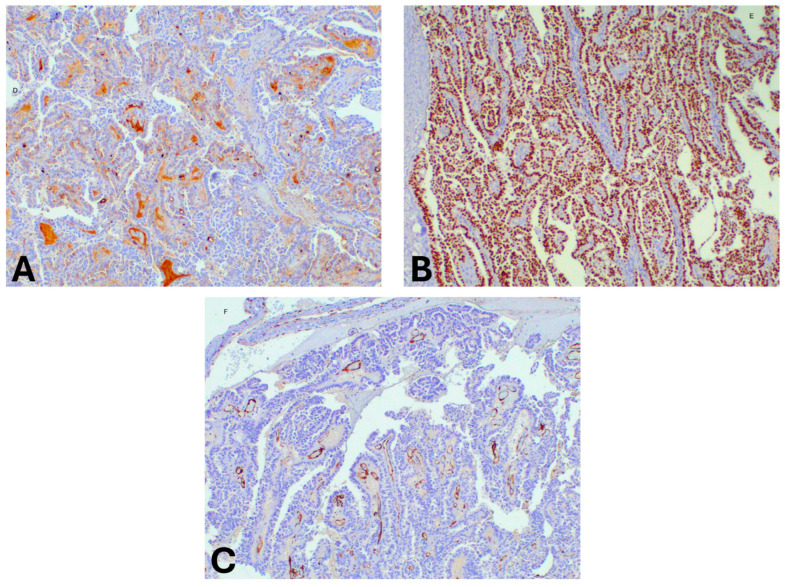
Immunohistochemical staining profile (all images at 200×): (**A**) tumor cells demonstrating strong cytoplasmic positivity for thyroglobulin; (**B**) nuclear co-expression of TTF-1 and PAX-8, confirming thyroid follicular epithelial origin; and (**C**) negative WT-1 staining, excluding a gynecologic or mesothelial primary.

**Figure 6:**
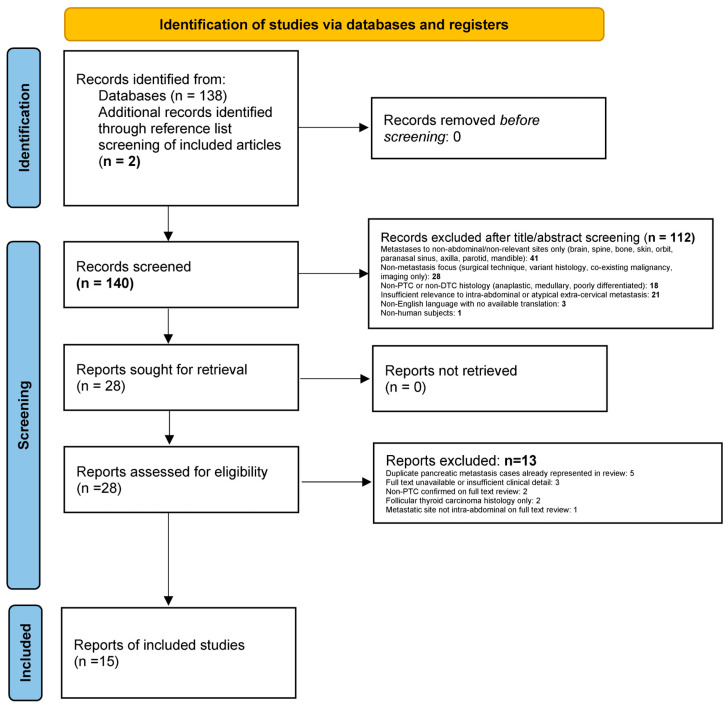
PRISMA flow diagram illustrating the search and selection process for the narrative review of intra-abdominal and atypical extra-cervical metastases from papillary thyroid carcinoma.

**Table 1:** Summary of reported cases of intra-abdominal and atypical extra-cervical metastases from papillary thyroid carcinoma, including metastatic sites, interval from primary diagnosis, treatment modalities, and clinical outcomes, highlighting the heterogeneity in presentation and management. Abbreviations: PTC, papillary thyroid carcinoma; RAI, radioactive iodine; EUS-FNA, endoscopic ultrasound-guided fine-needle aspiration; TSH, thyroid-stimulating hormone.

Author (Year)	Metastatic Site(s)	Interval from Primary Diagnosis	Treatment Modality	Reported Outcome
Angeles-Angeles et al. (2009) [11]	Pancreatic tail (large mass mimicking a primary pancreatic neoplasm); subsequent vertebral and brain metastases	Synchronous (primary tumor in an intrathoracic mediastinal thyroid; diagnosis delayed)	Distal pancreatectomy + splenectomy + partial colectomy; RAI therapy for 8 months	Poor response to RAI; disease progression with vertebral and brain involvement
Yang et al. (2019) [2]	Pancreatic body, liver surface, diaphragm (initial presentation mimicking pancreatitis); subsequent identification of bilateral parotid, bilateral salivary gland, left gluteus maximus, bilateral lung, right kidney, and multiple bone, rib, muscle, and subcutaneous metastases; BRAF V600E and TERT promoter mutation (C288T) confirmed	Synchronous (intra-abdominal metastases detected prior to thyroid cancer diagnosis)	Distal pancreatectomy + liver and diaphragm resection + cholangiojejunostomy for post-surgical obstructive jaundice; total thyroidectomy + bilateral central and lateral neck dissection + bilateral parotidectomy + bilateral salivary gland resection; RAI (no uptake in metastatic lesions—radioiodine-refractory); lenvatinib from December 2018	Serum thyroglobulin declined from 313.60 to 15.84 µg/L after 8 months of lenvatinib; patient alive with no apparent symptoms at the time of reporting
Cho et al. (2019) [12]	Pancreatic head/body (1.0 × 0.8 cm; diagnosed by EUS-guided FNA); concurrent left lower lobe and right upper lobe pulmonary metastases	10 years (with local recurrence at 3 years, treated with RAI and neck dissection)	Conservative wedge resection of lung nodule confirming metastatic PTC; pancreatic resection deferred due to systemic disease; systemic therapy planned	Systemic treatment initiated; local surgical approach to the pancreas to be reconsidered based on treatment response
Ligocka et al. (2019) [23]	Liver segment 7 (solitary 70 mm RAI-negative metastasis)	8 years	Anatomical right liver lobe resection; R0 resection achieved	No recurrence at 60-month follow-up; durable long-term disease control
Ren et al. (2020) [13]	Pancreas, liver, and diaphragm (synchronous; initially misdiagnosed as acute pancreatitis); additional metastases to bilateral parotid glands, bilateral submandibular glands, and cervical lymph nodes; BRAF V600E and TERT promoter mutations identified	Synchronous (detected at initial presentation prior to thyroid cancer diagnosis)	Distal pancreatectomy + splenectomy + liver and diaphragm resection + cholangiojejunostomy for post-surgical obstructive jaundice; total thyroidectomy + cervical lymphadenectomy + bilateral parotidectomy + bilateral submandibular gland resection; RAI therapy	Patient alive at the time of reporting; authors advocate an aggressive surgical approach in selected patients
Tramontin et al. (2020) [14]	Pancreatic head (presenting as cholestatic syndrome with jaundice and elevated liver enzymes)	6 years	Pancreaticoduodenectomy (Whipple procedure); sorafenib initiated postoperatively	Stable disease on sorafenib; patient asymptomatic at follow-up; Serum thyroglobulin remained elevated postoperatively
Wen et al. (2021) [15]	Solitary occult liver metastasis (right lobe; 9.2 × 6.5 × 2.5 mm; not visible on contrast-enhanced CT; detected only on post-ablation SPECT/CT; biopsy not feasible due to size)	Detected 1 month post-thyroidectomy	Two courses of RAI therapy (100 mCi then 120 mCi)	Radiologic regression, with the lesion reducing from 9.2 mm to 4.4 mm; Serum thyroglobulin declined from 0.25 to 0.01 ng/mL over 6-month follow-up
Yang et al. (2022) [16]	Concurrent liver metastases (multiple nodules up to 2.0 cm) and bilateral pulmonary metastases (multiple nodules up to 0.8 cm) from papillary thyroid microcarcinoma; possible tibial involvement on bone imaging	Synchronous (detected at initial presentation)	Total thyroidectomy + central neck dissection; I-131 therapy one year postoperatively	Recovered well postoperatively; pulmonary metastases showed no significant RAI uptake suggesting radioiodine-refractory disease; patient maintained on TSH suppression
Warda et al. (2022) [17]	Pancreatic head (tall cell variant PTC; detected 3 years after primary diagnosis in the setting of RAI-refractory disease; complex molecular profile including BRAF, KRAS, TERT, and RET/NTRK fusion events)	3 years	Radical pancreaticoduodenectomy (Whipple procedure); lenvatinib initiated postoperatively	Biochemical response with Serum thyroglobulin declining to 2.9 ng/mL; however, significant clinical deterioration with debilitating depression, weight loss, and palliative care involvement
Wang et al. (2013) [20]	Uterine corpus (large heterogeneous mass measuring 115 × 92 × 94 mm, mimicking ovarian cystadenocarcinoma; ovaries uninvolved)	Synchronous (detected on pre-operative imaging at time of thyroid cancer diagnosis)	Total thyroidectomy + total abdominal hysterectomy + bilateral salpingo-oophorectomy + omentectomy; patient declined further RAI therapy	No recurrence at 22-month follow-up
Song et al. (2023) [18]	Pancreatic tail (cystic mass with mural nodule measuring 5.6 × 5.0 cm; incidentally discovered during investigation for uncontrolled diabetes); bilateral pulmonary nodules managed conservatively	6 years	Distal pancreato-splenectomy with local mesocolon excision; BRAF V600E positive on histopathology; sorafenib planned if progression occurred	No recurrence at 3-year follow-up after pancreatic surgery; pulmonary nodules stable
Edamadaka et al. (2024) [22]	Urinary bladder (solitary sessile polypoid lesion measuring 2.8 × 1.9 cm; presenting as hematuria; thyroid primary incidentally detected on FDG PET/CT)	Synchronous (urinary bladder metastasis detected at initial presentation)	Transurethral resection of bladder tumor; total thyroidectomy; high-dose RAI therapy (150 mCi)	Iodine-avid disease confirmed on post-therapy SPECT/CT; follow-up outcome not reported
Thomasson et al. (2024) [24]	Liver segment 3 (presenting as spontaneous hepatic hemorrhage); subsequent vertebral, bladder, peritoneal, splenic, and pulmonary metastases	Synchronous (hepatic metastasis detected at initial PTC presentation)	Total thyroidectomy; left lateral liver segment resection; adjuvant RAI (iodine-refractory disease confirmed); lenvatinib; radiation therapy for vertebral metastases	Extensive disease progression despite multimodal therapy; patient died 22 months after initial presentation
Suliman et al. (2025) [21]	Bilateral inguinal lymph nodes (presenting as bilateral inguinal lymphadenopathy; 1 cm occult thyroid primary detected only after inguinal biopsy confirmed PTC)	Synchronous (inguinal metastases detected prior to thyroid primary identification)	Incisional biopsy for diagnosis; total thyroidectomy recommended but declined by patient due to cultural beliefs	Patient died approximately two months after declining surgical treatment; cause of death undocumented
Lopes et al. (2025) [19]	Pancreas (uncinate process; cystic lesion with mural nodule); retroperitoneal lymph node; vertebral and subsequent liver metastases	11 years	Pancreatic resection deferred due to multi-organ disease; stereotactic body radiotherapy; external beam radiotherapy for bone metastasis; denosumab; second-round RAI (no iodine-avid uptake); supportive care	Disease progression with liver metastases and cholangitis; patient died 23 months after pancreatic metastasis diagnosis

## Data Availability

All data generated or analyzed during this study are included in this published article. No additional datasets were generated.
